# Mesotherapy: From Historical Notes to Scientific Evidence and Future Prospects

**DOI:** 10.1155/2020/3542848

**Published:** 2020-05-01

**Authors:** Massimo Mammucari, Enrica Maggiori, Domenico Russo, Chiara Giorgio, Gianpaolo Ronconi, Paola E Ferrara, Flora Canzona, Luciano Antonaci, Bartolomeo Violo, Renato Vellucci, Domenico Rocco Mediati, Alberto Migliore, Umberto Massafra, Barbara Bifarini, Fabio Gori, Massimo di Carlo, Stefano Brauneis, Teresa Paolucci, Piergiovanni Rocchi, Anna Cuguttu, Raffaele Di Marzo, Alessandro Bomprezzi, Stefania Santini, Manuela Giardini, Anna Rosa Catizzone, Fiammetta Troili, Dario Dorato, Alessandra Gallo, Costanza Guglielmo, Silvia Natoli

**Affiliations:** ^1^Primary Care Unit ASL RM 1, Rome, Italy; ^2^“San Marco” Hospice and Palliative Care, Latina, Italy; ^3^Rehabilitation Unit, F Pirinei Hospital, Altamura, BA, Italy; ^4^Physical Medicine and Rehabilitation Unit, IRCCS, Catholic University of Sacred Heart, Rome, Italy; ^5^Istituto Dermopatico Dell'Immacolata, IRCCS Foundation, Rome, Italy; ^6^Pain Therapy Unit, S. Spirito Hospital, Rome, Italy; ^7^Palliative Care and Pain Therapy Unit – Anesthesiology and Resuscitation Department of Careggi University Hospital, Florence, Italy; ^8^Unit of Rheumatology, San Pietro Fatebenefratelli Hospital, Rome, Italy; ^9^Section of Anesthesia, Intensive Care, and Pain Medicine, Azienda Ospedaliera-Universitaria Santa Maria Della Misericordia, Perugia, Italy; ^10^Pain Therapy Unit, S Pertini Hospital, Rome, Italy; ^11^Pain Center “Enzo Borzomati”, University Hospital of Rome “Policlinico Umberto I”, Rome, Italy; ^12^University G. D'Annunzio Chieti, Department of Medical and Oral Sciences and Biotechnologies, Chieti-Pescara, Italy; ^13^Member of the Italian Society of Mesotherapy, Rome, Italy; ^14^Department of Clinical Science and Translational Medicine, Tor Vergata University, Rome, Italy

## Abstract

Intradermal therapy, known as mesotherapy, is a technique used to inject a drug into the surface layer of the skin. In particular, it involves the use of a short needle to deposit the drug in the dermis. The intradermal microdeposit modulates the drug's kinetics, slowing absorption and prolonging the local mechanism of action. It is successfully applied in the treatment of some forms of localized pain syndromes and other local clinical conditions. It could be suggested when a systemic drug-sparing effect is useful, when other therapies have failed (or cannot be used), and when it can synergize with other pharmacological or nonpharmacological therapies. Despite the lack of randomized clinical trials in some fields of application, a general consensus is also reached in nonpharmacological mechanism of action, the technique execution modalities, the scientific rationale to apply it in some indications, and the usefulness of the informed consent. The Italian Mesotherapy Society proposes this position paper to apply intradermal therapy based on scientific evidence and no longer on personal bias.

## 1. Historical Notes

The injections into the skin for therapeutic purposes date back to ancient Chinese and Indian medicine. More recently (Table 1), Karl Baunscheidt in 1847 convinced that a drug could act even if superficially injected and experienced dermal injection at a depth of two millimeters. In 1853, Alexander Wood (a Scottish physician) injected the first dose of dermic morphine to induce relief in many painful conditions. In 1860, Bartolomeo Guala began practicing the systematic hypodermic treatment in a hospital, and in 1867, Gaetano Primavera in Naples carried out the first experiment to assess the degree of drug absorption in the urine after hypodermic administration. In the same year, the London Medical Society, citing hypodermic injections, wrote “the speed, intensity and safety of the action, the production of a given effect with a lower dose of the other administrations, the certainty of the effects, the ease of application, the absence of certain disagreeable actions of other drugs.” In 1870, during the Franco-Prussian war, doctors injected distilled water into the dermis to relieve arthritic pain. In 1885, William Halsted reported that intradermal inoculation of sterile water induces local anesthesia. In 1894, Pietro Orlandini, a Venetian doctor, proposed dermal punctures for the treatment of some forms of localized pain, and in 1941, George D. Gammon and Isaac Starr published the analgesic effect of sterile water inoculation into the skin over or in the proximity of the pain. In 1958, Michel Pistor proposed the term “mesotherapy” to indicate the inoculation of drugs in the thickness of the skin. In 2004, Sergio Maggiori, analyzing preclinical and clinical trials, proposed the term “local intradermal therapy” (LIT) to emphasize that superficial inoculation allowed to reach the clinical effect with a lower dose of drug.

To date, LIT is one of the best known and most widely applied microinvasive techniques in many parts of the world for the treatment of various local clinical conditions. Over the past few years, we have noticed that very often patients ask a question: how does mesotherapy work? In order to answer this question, we propose a position paper by the Italian Society of Mesotherapy.

## 2. Rationale to Use Mesotherapy

Currently, local intradermal therapy (mesotherapy, LIT) is based on the hypothesis that the drug administered in the superficial layer of the skin allows a longer pharmacological action in the inoculation area and beyond. Preclinical studies have shown that the intradermal inoculation of anti-inflammatory [1], anesthetic [2], and antibiotic [3] allows a reduced dose and provides a prolonged maintenance in the tissues underlying the inoculation site (skin, muscle, and joint) as compared to intramuscular administration. Moreover, after intradermal injection of an antigen, a greater antibody response is obtained compared to intramuscular administration [4] suggesting that a lower dose inoculated into the dermis can achieve a greater effect than a deep inoculation. This technique modifies the normal kinetics of absorption of the injected drug; in particular, it slows systemic absorption and allows a distribution in the tissues underlying the inoculation site. The slow local spread and the longer persistence of the drug in the underlying tissues (up to the underlying articulation) allow the use of a lower dose of drug and a lower frequency of administration as compared to the systemic route [5]. The drug-sparing effect as compared to the systemic route, the possibility of treating patients already taking other pain killers [6], and the potential synergy with other pharmacological and nonpharmacological techniques [7] have allowed the rapid spread of this technique in many countries of the world. We have pointed out that in preclinical studies, an intradermal inoculated drug can diffuse in the underlying tissues maintaining tissue concentrations for longer periods of time than the intramuscular one [1]. These observations have led some researchers to study the effect of the intradermal therapy in patients with localized pain syndromes [3].

## 3. Technique

**Figure 1:**
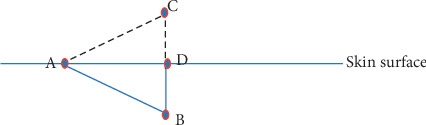
AB represents the needle with a length of 4 mm. AB inserted with an inclination of 30° constitutes one side of an equilateral triangle (ABC). AB = 4 mm; BD = 2 mm.

**Figure 2:**
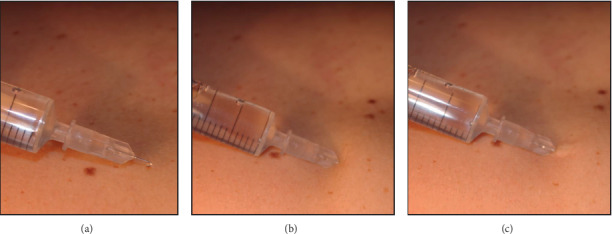
The technique: 30° inclination before the injection (a); the needle enters the dermis (b); liquid inserted superficially into the dermis—the whitening area shows the wheal (c).

**Figure 3:**
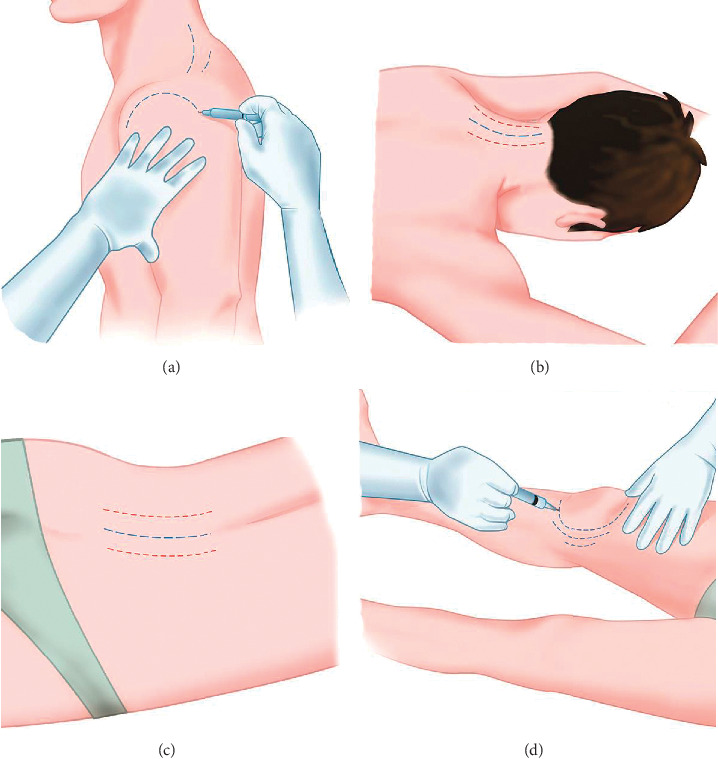
Some areas that can be treated: shoulder (a); cervical spine (b); lumbar spine (c); knee (d). The lines indicate the areas where to inoculate. The red and blue lines suggest two different inoculation pathways when different drugs are needed.

The mesotherapeutic technique (LIT) involves the inoculation of a drug with a 4 mm (27 Gouce) or 13 mm (30–32 Gouce) needle appropriately inclined to perform a microdermal deposit. According to some studies, the depth of intradermal injection could be 1 to 1.5 mm [8, 9] with individual variations. The most important variation in depth of the derma depends on body areas and age [10]. Dermal thickness increases linearly with age up to 20 years and decreases linearly with age subsequently [11]. Furthermore, it has been shown that women have a lower dermal thickness [9, 11]. Some authors reported significant differences only in some areas related to gender, BMI, and age [9]. Based on these individual variations, it could be difficult to standardize an intradermal inoculation. For this reason, we suggest a personalized needle inclination depending on the patient and the body to be treated. There are no randomized studies to compare the efficacy of inoculation in the different layers of the dermis (superficial dermis or deep dermis). Therefore, we suggest tilting the needle of thirty degrees to inoculate in the dermis without affecting the subcutaneous layer (Figures 1 and 2). The inclination of the needle also depends on the area to be treated and the thickness of the dermis (Figure 3). The technique requires medical and pharmacological knowledge and must be applied in compliance with rules of disinfection (appropriate disinfectants are needed), with sterile single-use devices, and in appropriate environment (Table 2).

## 4. Analgesic Mechanism of Action

Intradermal therapy has been administered to many patients with different types of localized pain (spinal, joint, muscle, tendon, etc.). Nevertheless, the available studies do not allow a standardization of this technique due to the different research methods. Significant clinical benefits such as pain control, improvement of quality of life, systemic dose reduction, and patient satisfaction were reported [7, 12, 13]. Given that mesotherapy is based on the inoculation of drugs through multiple microinjections, it is possible that the efficacy recorded in the experimental observations is due not only to the local effect of the drug but also to the action of the needle, or to the combination of both.

Costantino et al. in a randomized study evaluated the effects of LIT compared to a systemic treatment (oral and intramuscular) in patients with acute low back pain [14]. This study showed a similar analgesic effect in the two groups, with a lower dose of drugs consumed in the group treated with intradermal therapy. In another randomized trial, Saad reported a better outcome in terms of efficacy and quality of life in patients with chronic low back pain treated with LIT compared to treatment with oral anti-inflammatory drugs [15]. Chen [16] and Saggini [12] found significant improvement in the physical function, lower consumption of analgesics, and less adverse events compared to oral treatment. Kocak [17] conducted a randomized trial to evaluate the effects of LIT treatment compared to intravenous administration of anti-inflammatories. He found that patients treated in an emergency room for musculoskeletal pain reported significant better pain control after a single mesotherapy treatment. Yang reported a better analgesic effect after a single intradermal administration compared to oral NSAID administration in patients with localized pain [18]. These results suggest that drug-based intradermal therapy induces significant benefits in many localized pain syndromes with less medication and lower risk of adverse events.

However, a different efficacy has been reported in response to different analgesic drugs [19, 20] probably due to the different pharmacological potency and/or to the different capacity to remain longer in the underlying tissues. In addition to the local pharmacological effect, we must take into account the stimulus caused by micropunctures [21]. Comparing two groups of patients treated with anesthetic-mesotherapy or needle puncture alone (dry mesotherapy), it was found that both treatments induce pain control, even though anesthetic mesotherapy on trigger points was more effective [22]. These data suggest a synergistic effect between the local pharmacological action and a reflex analgesic action stimulated by the needle.

We also underline that the local analgesia is partly due to the effect of the inoculated liquid that causes distension of the dermis and local chemical variations. Three studies have reported that saline-based mesotherapy can reduce pain, although to a lesser extent and for less time, than drug-based mesotherapy [23–25]. It should be noted that the physiological solution injected superficially into the skin is less effective than sterile water for injections (SWI). In randomized studies, it was found that SWI is more effective than the physiological solution to manage lower back pain in women during childbirth [26, 27]. This greater effectiveness has also been demonstrated in a randomized study comparing SWI-based mesotherapy and dry mesotherapy [28]. The greatest analgesic effect of SWI could be explained by osmotic irritation and increased tissue pressure with consequent activation of afferent nerve fibers (A-delta and C fibers) and of gate control [29].

**Figure 4:**
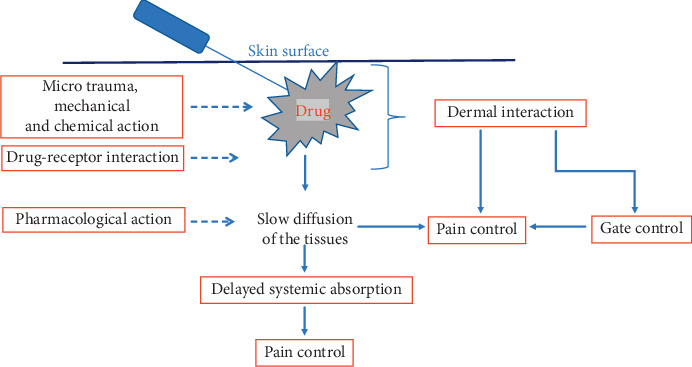
Possible mechanism of action. The drug (liquid) injected could stimulate the dermis and trigger a series of local and systemic reactions that participate in the control of pain. The dermis could contribute to the analgesic effect through a mediated mesodermal modulation of the intradermal glial cell system.

Generally, the drugs most commonly used to reduce localized pain in published studies are anesthetics, muscle relaxants, analgesics, and anti-inflammatory, alone or in combination [5–7], but their central mechanism of action does not explain the analgesic effect obtained with the local inoculation. Probably, drugs have been effective in interacting with the endorphin system and the peripheral immune system [30]: many localized pain syndromes are based on a kind of inflammation inducing an upregulation of endorphin receptors [31] and these systems could be the peripheral target of many analgesic drugs. We also point out that the inoculation site could play an analgesic role, as demonstrated by some authors who reported the effects of inoculation on trigger points in patients with chronic spinal pain [22, 32]. Any way, the local pharmacological effect, the needle-induced microinjections, the mechanical-chemical stimulation induced by the volume of liquid injected, and the stimulation of superficial trigger points do not explain some lasting effects over time obtained with intradermal therapy. But the recent discovery by Abdo [33], who identified the glial cells organized in a mesh network in the thickness of the dermis and their ability to control pain through the direct connection with sensory neurons, could explain why the mesotherapy technique produces all these encouraging results. We can hypothesize that the first rapid analgesic effect, often observed even after a single intradermal therapeutic application, and the medium- and long-term effects induced by LIT are the result of microinjections, of the mechanical-chemical stimulus induced by the injected liquid and of the local pharmacological action, but also the result of a series of complex interactions between the intradermal technique and dermal pain control systems (Figure 4). The dermis and, in particular, the glial cells could be the new potential target of injected drugs through the mesotherapy treatment.

More studies are needed to investigate the role of this technique in various forms of localized pain: acute or chronic, inflammatory or mechanical, nociceptive or neuropathic, and with or without degenerative genesis. Given the complexity of the mechanisms that regulate pain and the individual variability of the response to analgesic therapies, we strongly recommend that LIT must be applied based on the individual patient's condition. Applied for analgesic purposes, it allows three potential advantages: it induces a useful drug-sparing effect specially when a lower possible dose of medication is indicated for the patient (elderly, many concomitant diseases, high risk of drug interactions, etc.); it is useful when other therapies have failed or cannot be used; it synergize with other pharmacological or nonpharmacological therapies.

It is known that the intensity of needle-induced pain and the precision with which the needle is injected into the trigger point are related to the analgesic effect [34], but we strongly discourage to base the decision to repeat the treatment if the patient does not respond within the first three sessions. In many studies, most patients were treated with a number of sessions ranging from one to eight depending on the study protocol. In real practice, we suggest applying a standard algorithm based on the patient's response [7] even because in pain management, the treatment must always be tailored to the individual patient's response.

## 5. Combination with Other Therapies

Intradermal therapy can also synergize with other therapeutic strategies, for example, to reduce dose of systemic opioid [35] or to improve the effects of rehabilitation, in combination with ultrasound [36] or antalgic electrotherapy [37, 38]. It has a remarkable success even in patients undergoing rehabilitation programs for musculoskeletal disease [13] or after sports trauma [7]. Although laser and intradermal therapy have different frequency, it may be useful to consider a combination of these two therapies. It is possible to hypothesize that lasers can be used: (a) before LIT, to reduce discomfort by needles and to synergize with drugs injected; (b) after LIT, to maintain the analgesia result obtained. Nevertheless, we underline that to date, we have few clinical data to support combination of laser and mesotherapy [39, 40]. For this reason, their combination must be supported by *ad hoc* clinical studies in order to confirm the clinical rationale and identify tolerability and the best path of care for patients.

The slow diffusion, tissue pharmacological action, and interactions between needle-induced microtrauma and tissues may also be useful in other clinical forms. For example, in chronic venous disease, the functional morphological alterations induced by the microcirculatory alteration, chronic edema, and fibrosclerosis could benefit from local treatment. Indeed, improvement of edema, pain control, ultrasound appearance, and satisfaction of patients have been reported in patients with chronic venous disease of the lower limbs treated with LIT [41, 42]. These results could be interpreted as rational to treat the edematous fibrosclerotic panniculitis (EFP) induced by microcirculatory dysfunction of the subcutaneous tissue, hypoxia, and local dystrophic phenomena of the dermis [43]. For this reason, we recommend considering the combination of systemic treatment and LIT to slow the course of chronic venous disease. Many other skin alterations could benefit from the intradermal technique, such as alopecia [44, 45] and the low-grade of inflammation associated with skin aging [46]. We strongly suggest that a clinical diagnosis is essential before applying LIT. In fact, from the diagnosis derives the choice of the treatment path, and consequently, it will be possible to decide whether the “mesotherapy” is a first or second choice, alone or in combination with other therapies, and will also take into account the risk assessment, satisfaction of the patient, and resource consumption.

## 6. Tolerability and Safety of the Technique

Microinjections induced by mesotherapy cause the formation of a microdrug deposit that disappears within a few minutes. Local reactions of mild and short duration may occur, linked in part to the microtrauma produced by the needle and in part to the chemicophysical and biochemical activity of the drug which may generate transient erythema. Usually, the patient complains of a slight pain following the needle puncture, and even if they disappear within a few moments, the patient should be alerted and prepared for this reaction.

In the literature, cutaneous infections are reported mainly after treatments for aesthetic purposes [47]. However, the reported adverse events are mainly due to noncompliance with hygiene rules and to the practice of mesotherapy in an inappropriate environment or by nonprofessional personnel. We also do not recommend self-administration by the patient. The simplicity of this technique is only apparent, and both Health Authorities and manufacturers of injectable compounds should responsibly advise against any form of self-administration of substances in the skin for curative or preventive purposes.

## 7. Open Questions for the Next Clinical Research

Some questions remain open and will guide future clinical research. Many drugs with potential useful mechanism of action have been used, but no comparative studies exist to understand which of them is most effective. The choice of the principles to be injected depends on the clinical-instrumental diagnosis, as well as the modality of application of the technique, and doctors must manage the evaluation of the effects over time. Inoculations of herbs and mixtures of substances are known, sometimes based on the personal conviction of efficacy and sometimes suggested by pharma companies. Taking into account that even with the physiological solution or SWI have obtained therapeutic results, the scientific and ethical problem in the use of substances arises which have not demonstrated their effectiveness in a comparison study. For this reason, we strongly suggest to explain the patient the rationale of the use of each inoculated compound. The treatment of many diseases, including localized pain, involves multimodal treatment. Therefore, intradermal therapy should be considered only one of the pharmacological weapons available and it should be applied when a potential benefit is expected in the individual patient's pathway of care. Since the intradermal route is not without risk, we do not recommend drug mixtures in the same syringe, in particular, when there is not in vitro/in vivo evidence of stability and tolerability of mixtures. There is no evidence that any mixture retains the stability and effectiveness of the individual components, especially when more off-label drugs are mixed [5, 48]. Furthermore, in case of an allergy, it is not possible to identify which of the individual drugs is responsible for the reaction. Although modest data exist to reduce the risk of physicochemical interactions between drugs [49], some studies have reported tolerability and efficacy with drug combinations (analgesics, anesthetics, muscle relaxants, etc.). More studies are needed to compare the combination with the single drug. Therefore, we strongly suggest that the use of more drugs in the same syringe should be reserved in a protected medical environment and practiced only by qualified doctors. Although local diffusion is clinically proven [50], we cannot assume that it is identical for all drugs, and even if the superficially inserted needle (2 mm) allows a medium-term analgesic response [51], it is not possible to predict the response in each patient. Finally, given the complexity of the skin, it is the task of research to help us understand when it is more useful to inject into the superficial dermis and when into the deep dermis.

## 8. Ethical and Legal Aspects

Mesotherapy is a medical act that can only be practiced by medical personnel; otherwise, it must be assumed (in the Italian legal system) the crime of abusive exercise of the medical profession, provided for and punished by the Penal Code. One of the main responsibilities of the doctor is the informed consent. Informed consent is crucial to establish a proper patient medical partnership, and it must not be interpreted as a bureaucratic act, but an integral part of the care path focused to respect the patient [52]. Some barriers (religious or cultural) could emerge that hinder the full understanding of the value of informed consent. For example, in some cultures, family participation has greater value than individual consent. In any case, the doctor has the task of spreading the high ethical value that informed consent represents.

Given the complex structure of the LIT, where the doctor is also involved in delicate choices of environment of suitable drugs to be inoculated appropriately, it seems very important that the guidelines are issued as soon as possible, or that, in any case, the good clinical and welfare practices should be proposed by the scientific societies. We also highlight the economic sustainability of the mesotherapy technique in pain therapy. Obtaining the same results on pain using lower doses of drug results in a lower cost for the health system. Therefore, the use of the mesotherapy technique in pain therapy allows increasing the number of patients with the right of pain relief. Health Authorities should consider this ethical aspect.

## 9. Future Prospects and Developments

Some authors have recently suggested that routine use of mesotherapy in the emergency department be conducted [53]. It might be interesting to evaluate the value of intradermal therapy with a heath technology assessment program in different care settings. Health Authorities and pharmaceutical companies should consider the social value of drug savings through the intradermal route. Scientific societies should suggest guidelines for individual patient care.

## 10. Conclusions

For many years, we have believed that the therapeutic effect obtained depends particularly on the action of the injected drug and that more combined drugs can achieve greater efficacy. However, the data available today suggest that various mechanisms, besides the local pharmacological one, participate in the effect of this technique. So even apparently inert substances can induce short-term and medium-term responses. We suggest that the local spread of the drug and its biological effect, the chemicophysical properties of the inoculated liquids (e.g., osmolarity and pH), the needle-induced stimulus, the injection points, and the superficiality of insertion could interact with dermal mechanisms and induce the effects observed in clinical studies.

Probably, the intradermal therapy could also become a new experimental method to study interactions between drugs and skin-based mechanisms. Those who practice this technique, by correctly selecting the patients to be treated, with the aim of reducing localized pain and reducing the doses of systemic drugs, discover its therapeutic value. Both the GPs and the specialists could take advantage of this technique to facilitate the treatment path of many patients. The scientific questions still open must represent the goal of the research, not the excuse to discard the hypothesis: if a medicine works, but we do not know why, we should not refuse it.

## Figures and Tables

**Table 1:** Main researchers of mesotherapy.

Karl Baunscheidt	The first drug dermal injection (two millimeters)	1847
Alexander Wood	First injection of dermic morphine	1853
Bartolomeo Guala	Systematic hypodermic treatment in hospital	1860
Gaetano Primavera	First experiment to assess the degree of drug absorption in the urine after hypodermic administration	1867
The London Medical Society	Definition of “hypodermic injections”	1867
Physicians during the Franco-Prussian war	Doctors injected distilled water into the dermis for pain	1870
William Halsted	Intradermal inoculation of sterile water induces local anesthesia	1885
Pietro Orlandini	Dermal punctures for pain	1894
George D Gammon and Isaac Starr	The analgesic effect of sterile water inoculation into the skin for pain	1941
Michel Pistor	Proposed the term “mesotherapy”	1958
Sergio Maggiori	Proposed the term “local intradermal therapy” (LIT)	2004

**Table 2:** Ten steps for a correct mesotherapy.

1. Wear disposable gloves
2. Prepare single-use needles and syringes, disinfectants, and cotton wool
3. Accurate disinfection of the skin surface to be treated
4. Warn the patient that a specific surface will be treated and take the (preferably) lying position
5. Prepare the drugs to be injected (avoid exposing them to heat and light)
6. Clean carefully the skin to be treated
7. Inject the therapy in selected points during the medical examination
8. Wait a few minutes before letting the patient stand up again
9. Dispose of medical waste in the appropriate containers
10. Complete the medical record with the treatment carried out
